# The Patient Reported Inventory of Self-Management of Chronic Conditions (PRISM-CC): testing for bias across patient characteristics and languages

**DOI:** 10.1007/s11136-025-04124-5

**Published:** 2026-01-06

**Authors:** Ingrid Olsson, George Kephart, Tanya Packer, Sabine Björk, Ulf Isaksson, Anna Nordström, Åsa Audulv

**Affiliations:** 1 Department of Nursing, Umeå University, Umeå, Sweden; 2 Department of Community Health and Epidemiology, Dalhousie University, Halifax, Canada; 3 School of Health Administration, Dalhousie University, Halifax, Canada; 4 Department of Epidemiology and Global Health, Umeå University, Umeå, Sweden; 5 Artic Centre, Umeå University, Umeå, Sweden; 6 Department of Medical Sciences, Rehabilitation Medicine, Uppsala University & University Hospital, Uppsala, Sweden; 7 School of Sport Sciences, UiT The Arctic University of Norway, Tromsø, Norway

**Keywords:** Chronic disease, Differential item functioning, Differential test functioning, Measurement invariance, Patient reported outcome measure, Self-management

## Abstract

**Purpose:**

Developed simultaneously in English and Swedish, the Patient Reported Inventory of Self-Management of Chronic Conditions (PRISM-CC) assesses patients’ perceived difficulty managing life with long-term health conditions. This study assessed the comparability of the PRISM-CC across sociodemographic groups, number of health conditions and language (English and Swedish).

**Methods:**

Differential item functioning (DIF) and differential test functioning (DTF) were analysed by age, gender, education level, and number of conditions using independent English and Swedish datasets. Language-based DIF and DTF were examined using pooled data. An iterative hybrid ordinal logistic regression approach was applied to identify potential DIF across the PRISM-CC’s seven domains. The impact of flagged items on total scores (DTF) was evaluated by comparing test characteristic curves.

**Results:**

Few items were flagged for potential DIF in the English, Swedish or pooled data, and only at low cutoff values. The impact of items with potential DIF on DTF was negligible.

**Conclusion:**

The absence of meaningful DIF and DTF in either the English or Swedish version of the PRISM-CC or between English and Swedish versions provides further support for the PRISM-CC as a valuable tool for assessing self-management ease and difficulty. These results also demonstrate the value of simultaneous development of instruments in two languages. Further evaluation of DIF is necessary in populations with greater self-management challenges, such as among people with severe disease burden.

## Plain language summary

Health providers and researchers often use questionnaires and surveys to help make decisions, such as who needs treatment or what kind of treatment they need. Misleading or wrong decisions can be made if the tools are not well designed. This study was done to test whether a new tool called the Patient Reported Inventory of Self-Management of Chronic Conditions works equally well for men and women, older and younger people, and those with different levels of education and different numbers of conditions. It also tested whether the tool worked well in English and Swedish. The tool has 36 questions answered by patients, with results showing seven areas that people might find hard to manage. Over 1500 people from around the world provided data for this study. The results show that this tool yields comparable results across different types of patients in either English or Swedish.

## Background

Health systems worldwide struggle to manage the increasing demands of long-term health conditions associated with population ageing and changing disease patterns [1–3]. Prevention, improved efficiency, and effectiveness of interventions are essential to meet this challenge. Improving individuals’ ability to better self-manage their long-term health conditions can make a substantial contribution [4], as self-management has been shown to improve quality of life and health outcomes [5–9] while simultaneously decreasing healthcare costs [10]. Supporting patient self-management has, therefore, become internationally recognized as an essential function of healthcare systems [11, 12].

Given the complexity and changing nature of long-term health conditions, and therefore self-management, patients require different support at different times and from different healthcare professionals [7, 13, 14]. Consequently, providing tailored self-management support requires assessment of individual self-management challenges to establish if and what type of self-management support is needed [5, 15, 16]. In the research context, comprehensive self-management assessment measures are needed to describe, facilitate, and evaluate self-management interventions [17–20]. Despite this growing emphasis, self-management practice and science have been hampered by lack of rigorously designed measurement instruments. Comprehensive and valid measures are critical for both research and clinical practice.

Unfortunately, there are widespread deficiencies in existing self-management measures [17–20]. They vary considerably in their aim, scope, theoretical foundation, and development [17, 19, 20], most are disease-specific, making them less suitable for individuals with multimorbidity [17, 20], and few acknowledge or measure the multi-dimensionality of patient self-management. Lack of suitable measures has necessitated use of related, but non-specific measures of quality of life [6, 21], activity level, or self-efficacy [17], complicating both interpretation and comparison of results [21].

To address these limitations, the Patient Reported Inventory of Self-Management of Chronic Conditions (PRISM-CC) was developed as a generic, multi-dimensional instrument that assesses perceived self-management ease or difficulty across seven self-management domains important for individuals living with one or more long-term health conditions [22, 23]. The PRISM-CC’s seven domains are based on a validated theoretical framework: the Taxonomy of Everyday Self-management Strategies (TEDSS). TEDSS measures five goal-oriented domains (Internal, Social Interaction, Activities, Healthy Behaviours, and Disease Controlling domains), and two support-oriented domains (Process and Resource domains) [24] (see Table 1). TEDSS is increasingly being used to describe self-management and self-management support [25–27]. Based on TEDSS, developed iteratively in both English and Swedish and adhering to current guidelines for developing patient-reported outcome measures [28–30], the PRISM-CC is scaled using a multi-dimensional item response theory (IRT) graded response model. Robust construct validity, internal consistency, and test-retest reliability have been established [23, 31, 32].

A crucial next step in the PRISM-CC’s validation is to examine whether it performs equivalently across demographic groups and languages. This involves testing for differential item functioning (DIF) and differential test functioning (DTF) to detect potential measurement bias [28]. Such bias could compromise clinical decision-making and research comparability across studies and groups. Because factors such as age [33, 34], disease progression [35], low levels of education [36, 37], and gender roles [35] are associated with self-management, it is essential that the PRISM-CC remains invariant across these characteristics. A valid measure should perform similarly regardless of these differences. Stated methodologically, DIF occurs when individuals with the same level of the trait being measured have systematically different response probabilities to an item depending on patient characteristics [38]. If one or more items in a measure display DIF, it may lead to DTF, resulting in systematic bias in test scores or variation in measurement error [39, 40].

This study assessed DIF and DTF within and between the English and Swedish versions of the PRISM-CC by age, gender, level of education, number of health conditions and language. Based on the rigorous development, refinement, selection, and translation process used in PRISM-CC item selection, we hypothesized that there would be no meaningful DIF or DTF within or between the English and Swedish PRISM-CC.

## Methods

### PRISM-CC Development

**Fig. 1:**
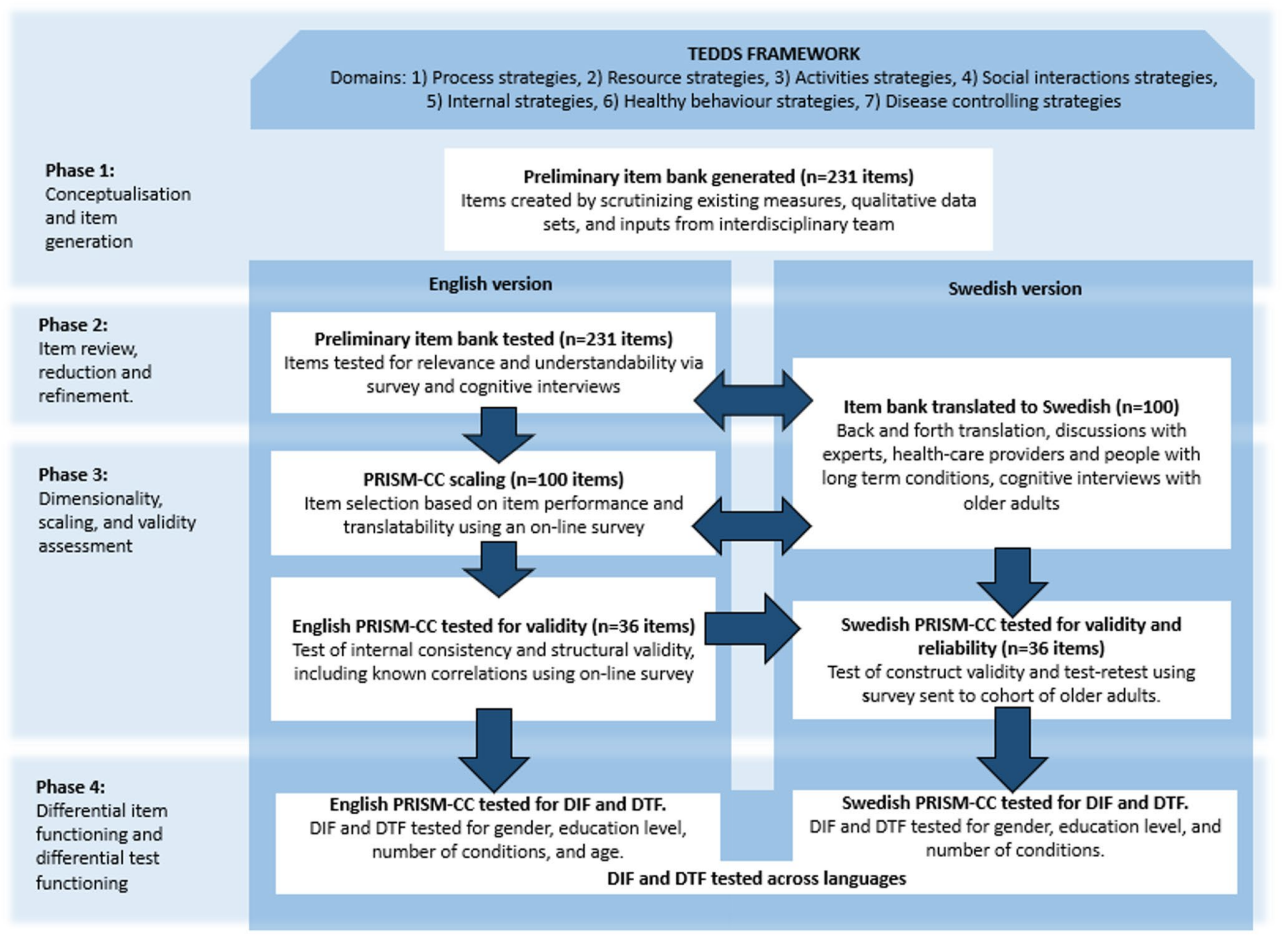
Overview of the PRISM-CC development process

The PRISM-CC was developed by a multilingual research team. With slight variation in sequence, development occurred simultaneously in English and Swedish, to promote cross-linguistic conceptual equivalence (see Fig. 1).

Item development and selection were also guided by multiple strategies designed to minimize differential item and test functioning (DIF/DTF) [23, 32]. Central to this process was the TEDSS framework, which provided a robust conceptual foundation for operationalizing self-management as perceived ease or difficulty within each domain [23] and provided conceptual guidance for item development and selection. In Phase 1, a preliminary item bank (231 items) was constructed using existing instruments and qualitative data derived from both English and Swedish studies. At each step, items were subject to rigorous face and content validity assessment. In Phase 2, the preliminary item bank was subjected to initial testing and translation to Swedish. Translation adhered to the Patient-Reported Outcome (PRO) Consortium translations process [41], including forward translation by a professional native speaking Swedish translator flueunt in English and back translation by a professional native speaking English translator fluent in Swedish, as well as discussions with an expert group. Some small edits of the translation was made to capture the essence of the items, while making them understandable in Swedish. Cognitive interviewes were performed in both languages [22, 31, 32], reducing the item bank to 100 items. These bilingual cognitive interviews provided critical insight into cross-language interpretation and potential sources of DIF, informing item refinement. In Phase 3, additional data collection, followed by psychometric testing, was used to select the final set of items. Then, the English version was scaled and assessed for structural and construct validity [23]. Swedish survey data were then used to assess the performance of the 36 items in Swedish and, in the case of one item showing suboptimal properties, select a replacement item. Structural validity and test-retest reliability were also tested for the Swedish version [31].

This paper reports the results of Phase 4: evaluating DIF and DTF of the PRISM-CC, thus were the English and Swedish datasets originally used for item selection, scaling, and construct validation reanalysed [23, 31].

### Study Data

The PRISM-CC was designed as a generic instrument for individuals with long-term health conditions; thus, both datasets employed broad inclusion criteria. However, the recruitment strategies and eligibility criteria differed between the English and Swedish datasets based on the research question and access to participants. Therefore, the English and Swedish samples differ not only by language but also in demographic, clinical characteristics and number of participants.

The English dataset was collected via an online survey promoted through patient organizations, healthcare settings, social media, and recruitment platforms [23]. Eligible participants were adults (≥ 18 years) with one or more self-reported long-term health conditions. In total, 1213 people completed the survey, 158 were excluded due to > 50% missing item responses, leaving 1055 participants for inclusion in this study. Data collection adhered to the Canadian Tri-Council Policy Statement on Ethical Conduct for Research Involving Humans (including informed consent); ethics approval was given by the Nova Scotia Health Research Ethics Board (Romeo File #: 1025263).

The Swedish dataset was collected in collaboration with the Healthy Aging Initiative (HAI). This population-based study invited all residents in the municipality of Umeå to participate the year they turned 70 years of age [42]. For this dataset, a follow-up survey was distributed to 1117 participants who had participated in the HAI study in 2018 and 2019 [31]. Since recent information about the individual’s health status was unavailable, the survey was sent to all enrolled participants, informing them that only those with one or more long-term health conditions were eligible and should complete the survey. Of the 1117 individuals invited, 542 respondents met the inclusion criteria of having one or more long-term health conditions. After removing individuals with > 50% missing item responses, 516 participants were included in this study. The HAI study received ethical approval from the Swedish Ethical Review Authority and the Regional Ethics Review Board in Umeå in 2007 (Dnr 2012-85-32 M and Dnr 07-031 M), and complementary ethical applications to add the PRISM-CC data collection and to pool the English and Swedish data were approved in 2020 (Dnr 2020–02387), and 2022 (Dnr 2022-05547-02) respectively. All participants gave informed consent.

The PRISM-CC is comprised of 36 items (4–8 per domain: see Table 1 for domain definitions): items have been described elsewhere [23, 31, 32]. All items are formulated as statements and have a six-option response scale indicating respondents perceived difficulty (ranging from ‘cannot do’ to ‘very easily’) or agreement with each item. Low scores indicate more self-management difficulty. A ‘not applicable’ category was also available. In addition to the PRISM-CC, sociodemographic characteristics (gender, marital status, highest education level and financial status), and the number and type of self-reported long-term health conditions were collected. Age was collected in the English survey, whereas the age of Swedish participants was limited to 72–73 years.

**Table 1:** The PRISM-CC domains and number of items

Resource (4 items)	Self-perceived ease or difficulty in seeking, pursuing and/or managing needed formal or informal supports and resources.
Process (5 items)	Self-perceived ease or difficulty in seeking information, being aware of choices and making good decisions.
Internal (8 items)	Self-perceived ease or difficulty in creating inner calm by preventing and managing stress, negative emotions, and internal distress.
Activity (5 items)	Self-perceived ease or difficulty in participating in everyday activities (leisure activities, work activities, household chores).
Social Interaction (5 items)	Self-perceived ease or difficulty in disclosing health issues, managing social interactions and relationships.
Healthy Behaviours (5 items)	Self-perceived ease or difficulty maintaining a healthy lifestyle in order to enhance health and limit the risk of lifestyle-related illness
Disease Controlling (4 items)	Self-perceived ease or difficulty in managing health conditions including managing medications and treatments, monitoring symptoms, and limiting complications.

### Statistical analysis

Stata version 18 [43] was used to analyse sample demographics, clinical characteristics, and item responses. Differential item (DIF) and test functioning (DTF) were assessed according to patient characteristics: age (only English data set), gender, education, number of reported long-term health conditions, and language. As the analysis required dichotomized variables, age was grouped in two ways to reflect comparisons between age groups with different life experiences and chronic disease patterns: 18–30 versus 31 and over, and 18–60 versus 61 and over. In both datasets, gender was dichotomized as female and male; education as lower education (incomplete elementary school, elementary school, high school) and higher education (bachelor’s degree, graduate degree); and number of conditions as 1–2 condition(s) or ≥ 3 conditions. Individuals with missing values for patient characteristic variables were excluded from associated analyses. As described in prior publications [23, 31], missing values due to item non-response and non-applicable responses in both the English and Swedish data were imputed using chained multiple imputation.

### Differential Item and Test Functioning

As the sociodemographic and long-term health condition attributes of the English and Swedish data were substantially different, we first assessed DIF/DTF separately in the English and Swedish data sets regarding age (English only), gender, education and number of conditions. We then used pooled data to assess DIF/DTF by language. To further assess whether DIF/DTF results by language reflect differences in demographics and chronic disease distributions, a sensitivity analysis was conducted with the English data restricted to > 40 yrs of age (*n* = 444) pooled with the Swedish data. This cutoff was chosen to focus on chronic conditions common in middle and older ages, while also ensuring adequate sample size.

DIF and DTF were assessed for each of the seven PRISM-CC domains by each patient characteristic (gender, level of education, two age groupings (English data), number of conditions, and language) using iterative hybrid ordinal logistic regression [44], implemented with R version 4.3.2 [45] and the “Lordif” R package version 0.3-3 [44]. This method assesses DIF by comparing three nested ordinal logistic regression models to determine whether a patient characteristic is associated with item response probabilities after adjustment for the latent variable (“theta”), represented by the estimated scores from the graded item response theory (IRT) model. The logic of this approach is that among subjects with the same value of theta, association of a patient characteristic with the probability of item response is evidence of DIF. The three ordinal logistic regression models are:

$$\:\mathrm{M}\mathrm{o}\mathrm{d}\mathrm{e}\mathrm{l}\:\left(1\right)\:\:P\left({u}_{i}\ge\:k\right)={a}_{k\:}+\:{\beta\:}_{1}\theta\:$$

$$ \begin{aligned} {\mathrm{Model}}\left( 2 \right)P\left( {u_{i} \ge k} \right) = & a_{k} + \beta _{1} *\theta \\ & + \beta _{2} *Group \\ \end{aligned}$$

$$ \begin{aligned} & {\mathrm{Model}}\left( 3 \right)P\left( {u_{i} \ge k} \right) = \\ & a_{k} + \beta _{1} *\theta + \beta _{2} *Group + \beta _{3} *\theta *Group \\ \end{aligned} $$

$\:P\left({u}_{i}\ge\:k\right)$ is the cumulative probability that an item response ($\:{u}_{i}$) falls in ordinal category *k* or higher, the $\:{a}_{k}$ are intercepts (i.e. thresholds) between the *k* categories and $\:{\beta\:}_{1}$ is the slope parameter for estimated scores for the latent variable ($\:\theta\:$). In model (2), $\:{\beta\:}_{2}$ measures the degree of “uniform” DIF by a patient characteristic, where the DIF is systematic across level of theta, while in model (3), $\:{\beta\:}_{3}\:$measures the degree of non-uniform DIF by a patient characteristic, where the DIF varies across levels of theta. Assessing improvements in fit between models assesses overall DIF (3 versus 1), uniform DIF (2 versus 1), and non-uniform DIF (3 versus 2). A problem with model estimation is that values of theta represented by IRT scores incorporate any DIF present in the items, thus biasing the estimation of DIF. To address this, the procedure uses an iterative “purification” process where estimates of theta are adjusted to correct for initial estimates of DIF, and then the analyses are repeated. This iterative process continues until the same set of items with evidence of DIF are identified [44, 46].

We used changes in McFadden pseudo-R² between the three ordinal logistic regression models as the criteria to detect items with DIF [47]. This is preferable to the use of likelihood ratio$\:\:{\chi\:}^{2}$ tests, in which sensitivity depends on sample size and the precision of scores from the graded response model [48], and the Mantel-Haenszel (M-H) technique, which cannot be used to assess non-uniform DIF [49]. There is debate and disparate guidance in the literature as to cutoffs for changes in R² indicative of significant DIF [47, 49–52]. Accordingly, we used progressively more stringent cutoffs. Initially, we used a cutoff of > 0.035 which has been used to differentiate negligible from moderate DIF and is more conservative than a criteria of > 0.13, which has also been used [53]. However, this cutoff did not flag any items as having DIF for any patient characteristic in either the English or Swedish data. We thus used even more conservative proposed criteria (> 0.01 and > 0.006) to flag items with potential DIF [49]. A concern with low cutoffs is the risk of Type I error (false-positive identification of DIF) [50]. However, this is not a concern since the goal was to flag items for potential DIF, rather than to select items.

The most important analysis focused on the assessment of DTF, since the magnitude of bias in final scores resulting from DIF matters when using a measure [39, 54]. The effect of item DIF on scores can be washed out, counteracted or compounded by the influence of other items. For all domains where items were estimated to have DIF, DTF was assessed using two types of plots [44]. First, to assess the magnitude of bias by patient characteristics, we plotted and compared test characteristic curves showing differences in the expected total scores by level of theta, for each patient characteristic due to DIF. In the absence of DIF, the curves would be identical, and differences between the curves show the magnitude of DTF due to DIF. Second, to assess the distribution of bias for individual scores resulting from DIF, we plotted the bias for each subject (measured as the difference between “naïve” scores that ignored DIF and “purified scores” that accounted for DIF) by “naïve” (unpurified) levels of theta. In the absence of clinical criteria for a meaningful level of bias, we classified meaningful bias due to DIF based on the 25th percentiles of the standard error of estimated theta values across the seven domains, which ranged from 0.24 to 0.33 in the English data and from 0.24 to 0.30 in the Swedish data. Accordingly, we conservatively defined meaningful bias as greater than 0.24.

## Results

As noted, because of differences in recruitment, the sociodemographic characteristics of the English and Swedish participants differed (Table 2). Compared to the Swedish subjects, English subjects were younger, more likely to be female, highly educated, and to report greater perceived impact of their conditions on daily life.

**Table 2:** Sociodemographic characteristics of the English and Swedish data

	Swedish total sample (*n* = 516)	Swedish female (*n* = 256)	Swedish male (*n* = 249)	English total sample (*n* = 1055)	English female (*n* = 719)	English male (*n* = 300)
Characteristic	n (%)	n (%)	n (%)	n (%)	n (%)	n (%)
*Age*						
18–30	0	0	0	354 (33.6)	222 (30.9)	112 (37.3)
31–60	0	0	0	328 (31.1)	238 (33.1)	78 (26.0)
> 60	516 (100)	256 (100)	249 (100)	268 (25.4)	184 (25.6)	83 (27.7)
Missing	0	0	0	105 (10.0)	75 (10.4)	27 (9.0)
Number of diseases*						
1–2 disease(s)	260 (50.4)	116 (45.3)	140 (56.2)	740 (70.1)	491 (68.3)	235 (78.3)
≥ 3 diseases	256 (49.6)	140 (54.7)	109 (43.8)	315 (29.9)	228 (31.7)	65 (21.7)
*Perceived impact of disease(s) on life*						
Extremely	24 (4.7)	13 (5.1)	11 (4.4)	260 (24.8)	181 (25.2)	69 (23.0)
Quite a bit	92 (17.8)	52 (20.3)	38 (15.3)	379 (36.1)	272 (37.8)	87 (29.0)
Moderately	176 (34.1)	90 (35.2)	81 (32.5)	290 (27.6)	193 (26.8)	91 (30.3)
A little bit	139 (26.9)	69 (27.0)	69 (27.7)	107 (10.2)	61 (8.5)	46 (15.3)
Not at all	73 (14.2)	26 (10.2)	44 (17.7)	11 (1.1)	6 (0.8)	5 (1.7)
Missing	12 (2.3)	6 (2.3)	6 (2.4)	8 (0.4)	6 (0.9)	2 (0.7)
*Level of education*						
Lower education	263 (51.0)	116 (45.3)	146 (58.6)	231 (21.9)	157 (21.8)	60 (20.0)
Higher education	235 (45.5)	136 (53.1)	99 (39.8)	815 (77.3)	557 (77.5)	236 (78.7)
Missing	18 (3.5)	4 (1.6)	4 (1.6)	9 (0.9)	5 (0.7)	4 (1.3)

Few items were flagged for potential DIF in either the English or Swedish data, and only at low cutoff values for changes in R^2^ (Table 3). In the English data, our analysis tested for uniform and non-uniform DIF in 36 items by four different patient characteristics. Despite the large number of tests, potential DIF was flagged for only three items across three domains (Social Interaction, Healthy Behaviours, Internal): two by gender and only one of the two tested age grouping (i.e. age dichotomized as 18–60 vs. 61 and over). However, the effect sizes were small, with two items flagged for uniform DIF based on changes in R^2^ slightly greater than 0.01 and an additional item flagged for uniform DIF with a change in R^2^ of 0.008. Somewhat more items were flagged for potential DIF in the Swedish data. With tests for 36 items across three different subject characteristics, nine items were flagged for potential DIF by gender, education level or number of health conditions. Again, all had small effect sizes, except for one item in the disease-controlling domain with a change in R^2^ of 0.02 for uniform DIF (Table 3).

**Fig. 2:**
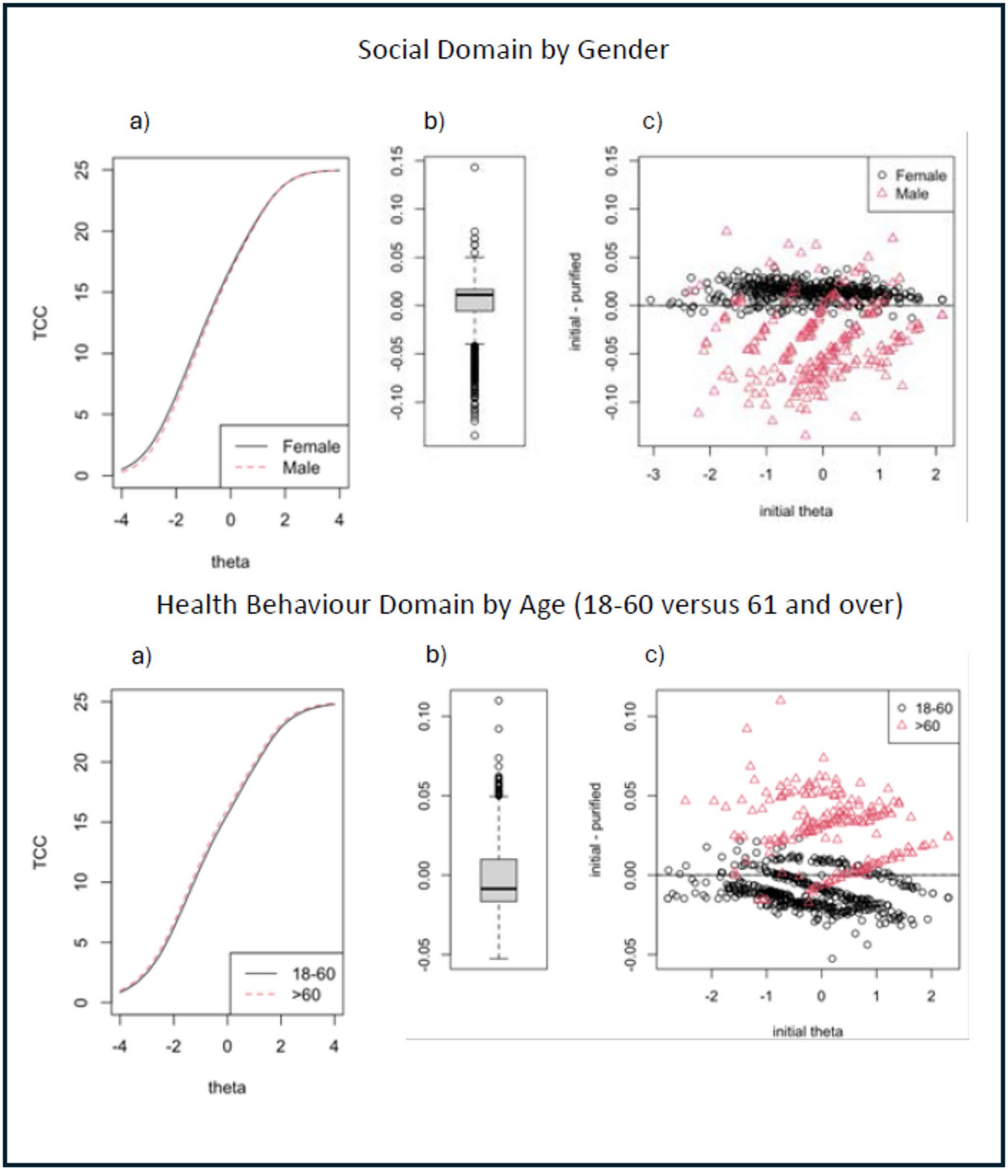
DTF analysis of bias for the two domains with the largest DIF in the English dataset. The left-hand panels (labelled “**a**”) show the difference in test characteristic curves (i.e. expected summative test scores by level of theta) due to DIF. The two right-hand panels (labelled “**b**” and “**c**”) show the estimated distribution of bias in individual scores due to DIF

**Fig. 3:**
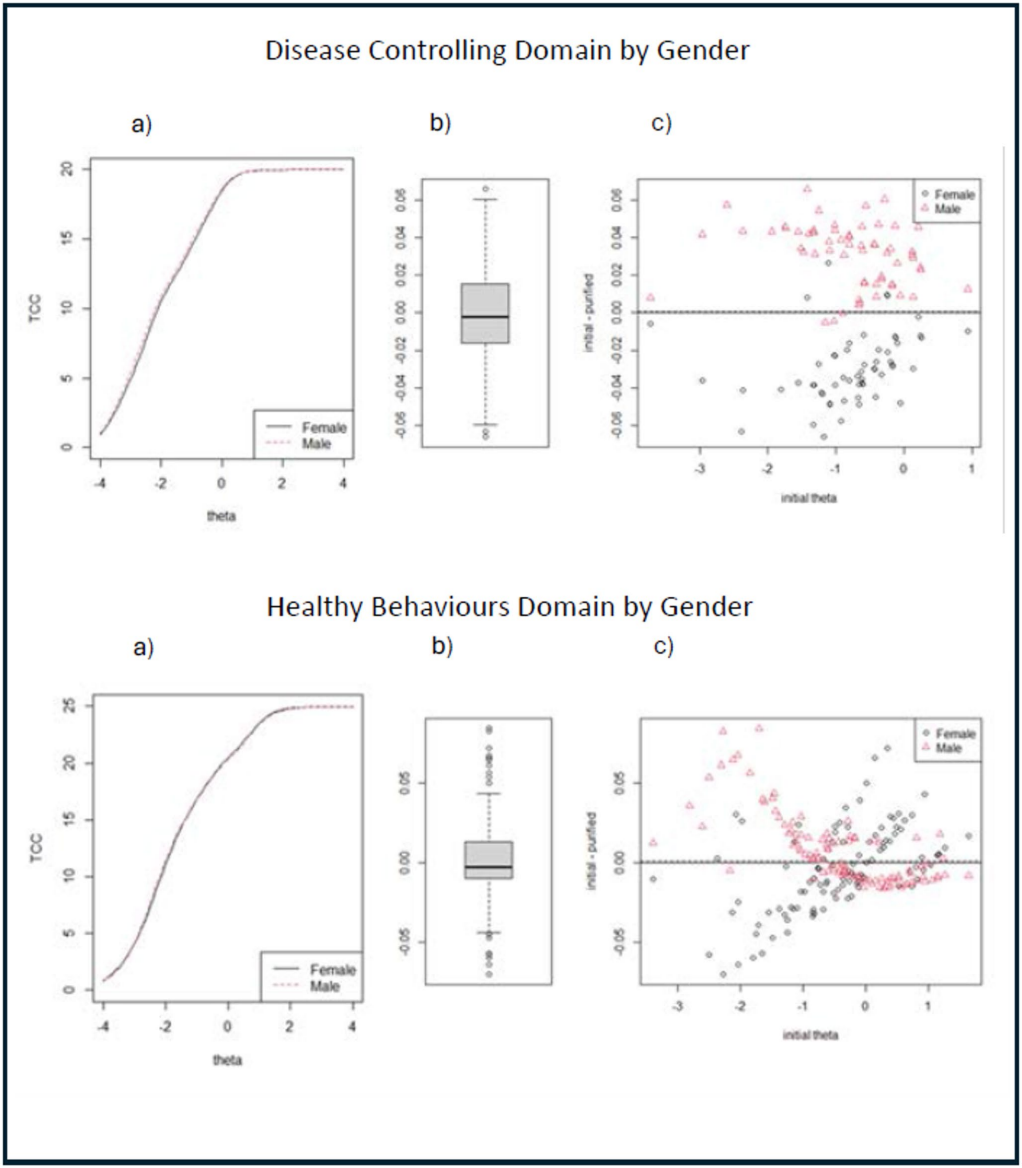
DTF analysis of bias for the two domains with the largest DIF in the Swedish dataset. The left-hand panels (labelled “**a**”) show the difference in test characteristic curves (i.e. expected summative test scores by level of theta) due to DIF. The two right-hand panels (labelled “**b**” and “**c**”) show the estimated distribution of bias in individual scores due to DIF

In both the Swedish and the English data, the estimated bias in measurement from items flagged for DIF (see Table 3), as assessed through DTF analysis, was very small. The items flagged for DIF contributed almost no difference in expected test scores across the full range of theta (see Fig. 2 English, and Fig. 3 Swedish). The box-whisker plots in the left panel of the figures show that: (1) in the English data (for both domains where items were flagged for potential DTF), the 10th and 90th percentiles of bias in subject scores were about ± 0.05; and (2) in the Swedish data, the 10th and 90th percentiles of bias were approximately ± 0.06 for the Disease Controlling domain and ± 0.04 for the Healthy Behaviours domain. These values, and the minimum and maximum biases in subject scores shown in the plots, are considerably below our cutoff for significant bias (0.24).

**Fig. 4:**
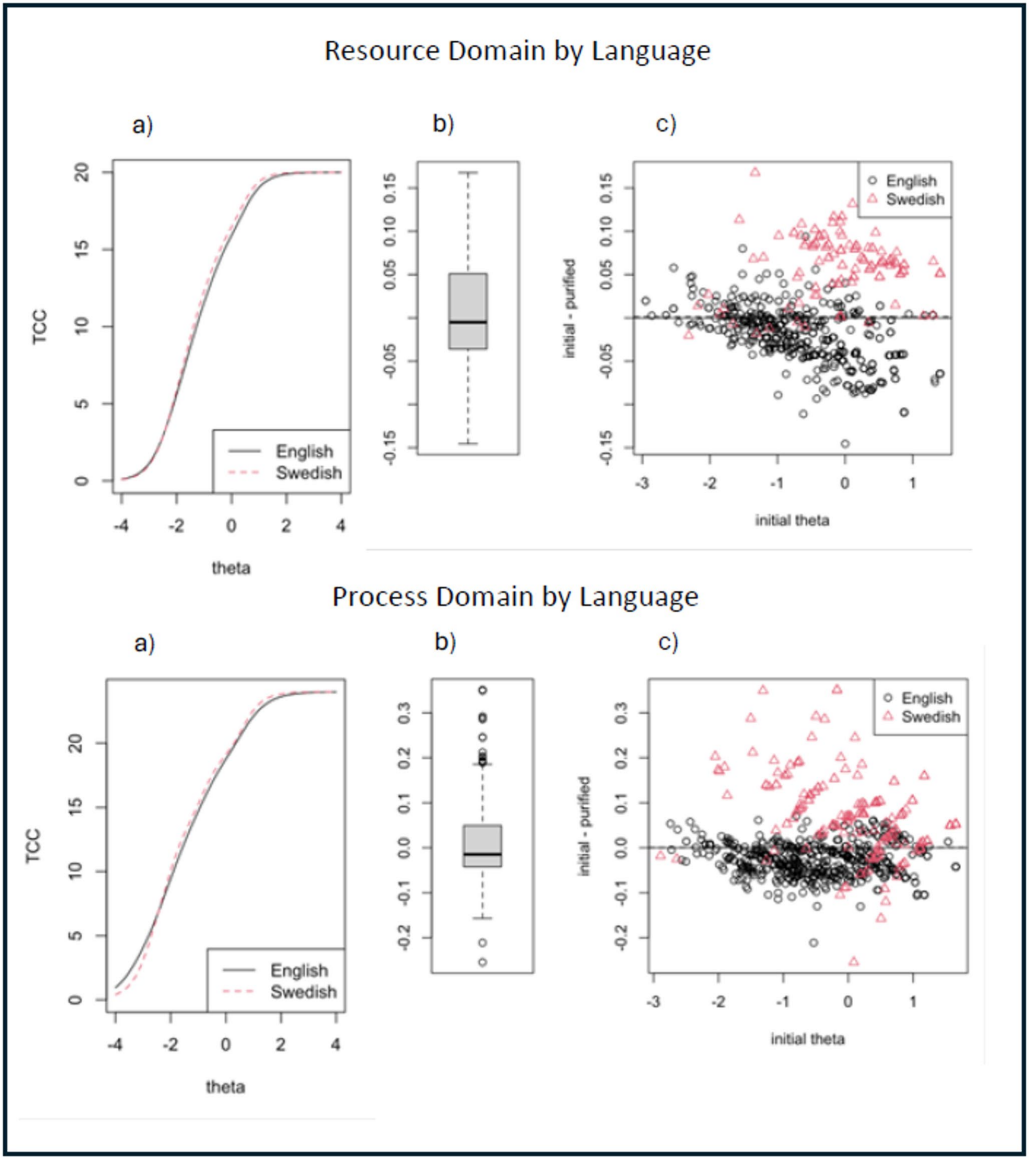
DTF analysis of bias for the two domains with the largest DIF across languages. The left-hand panels (labelled “**a**”) show the difference in test characteristic curves (i.e. expected summative test scores by level of theta) due to DIF. The two right-hand panels (labelled “**b**” and “**c**”) show the estimated distribution of bias in individual scores due to DIF

Having established negligible DIF and DTF in either the English or Swedish data, we proceeded to pool the two datasets and assess DIF and DTF by language. Only four items (across three domains) were flagged for potential DIF by language, two of which had changes in R^2^ indicating uniform DIF greater than 0.02. DTF analysis indicated negligible bias in domain scores resulting from DIF in these two items (Fig. 4). Bias in individual scores was greater than observed in the separate English and Swedish analyses (10th and 90th percentiles: ±0.15 for the Resource domain and − 0.15 to 0.19 for the Process domain), but were still below the 0.24 cutoff we established for meaningful bias. The sensitivity analysis, limiting the English sample to ages > 40, produced highly similar results to analyses using the full sample. The same items were flagged for potential DIF, and they contributed negligible bias to domain scores.

**Table 3:** Items flagged for potential Uniform, Non-Uniform and overall differential item functioning by variable and data source (English, Swedish and Pooled)

Domain		Item	Variable	Change in McFadden *R*²		
				1 vs. 2¹	2 vs. 3²	1 vs. 3³
*English data*						
	Social Interaction	Soc4: I devote time and attention to those who are dear to me	Gender	0.012	< 0.001	0.012
	Healthy Behaviours	Hea3: I find ways to train my brain to keep mentally fit.	Age^4^	0.011	< 0.001	0.012
	Internal	Int4: I have and use ways to recover after a bad day	Gender	0.008	< 0.001	0.008
*Swedish data*						
	Disease Controlling	Dis1: When problems with my health arise, I understand what to do to manage my condition(s).	Gender	0.020	< 0.001	0.020
	Healthy Behaviours	Hea5S: I maintain healthy behaviours even when I have a lot to do.	Gender	0.010	0.007	0.016
	Healthy Behaviours	Hea3: I find ways to train my brain to keep mentally fit.	Gender	0.015	< 0.001	0.015
	Process	Pro4: I try different things to find out what works best for me.	# Conditions	0.002	0.013	0.015
	Healthy Behaviours	Hea3: I find ways to train my brain to keep mentally fit.	Education	0.011	< 0.001	0.011
	Social Interaction	Soc5: When problems with my health arise, I stay in touch with people who are important to me.	Education	0.010	< 0.001	0.010
*Pooled data*						
	Resource	Res4: When I need to, I find people to help me understand information I receive about my condition(s)	Language	0.027	< 0.001	0.028
	Process	Pro4: I try different things to find out what works best for me.	Language	0.021	0.007	0.028
	Process	Pro2: I make informed decisions	Language	< 0.001	0.009	0.009
	Internal	Int7: I focus on the positives	Language	0.007	< 0.001	0.007

¹Uniform DIF, ²Non-uniform DIF, ³ Overall DIF, ^4^ Age grouped 18–60 versus 61 and over.Reported change in R² between models is after “purification” of latent variable for DIF. Within each data set, items are sorted by largest change in McFadden R^2^.

## Discussion

No evidence of meaningful DIF or DTF in the PRISM-CC by gender, level of education, number of conditions, age (18–60 vs. 61 and over) or language was found. The low level of DIF and DTF builds confidence in using PRISM-CC with diverse patient populations and in cross-language studies. Failure to appropriately assess patient-reported outcome measures for DIF and/or DTF before making group comparisons may lead to significant interpretational challenges, as differences can result from how the two groups interpret the latent construct and not a real group difference [54]. Evaluating a measure’s equivalence is crucial to ensure fair patient assessment [55]. Clinicians can be confident that use of the PRISM-CC is a consistent measure of self-management ease or difficulty, regardless of the age, gender, education or multimorbidity of patients. It can, therefore, be used to identify areas of concern and tailor care. Researchers can also be confident in comparisons between studies by variables assessed in our study and expect that comparisons by other variables are also unlikely to be biased.

As noted, a finding of DIF and DTF within a measurement tool identifies potential biases. In contrast, a finding of no or limited DIF or DTF strengthens the confidence in the tool’s results. The PRISM-CC stands out as one of only a few self-management measures assessed for DIF and/or DTF [56–58]. Where DIF has been assessed for self-management measures [59–66], and self-reported health measures more generally [57, 67, 68], DIF by gender, education and across disease groups have been found. The Patient Activation Measure [69, 70], one of the most widely used measures to assess self-management, has shown DIF across gender and education [59–63], and across different disease groups [64, 65]. DTF was also found for the Health Education Impact Questionnaire across disease groups in two of eight subscales [66]. Self-management measures not tested for DIF and/or DTF should be used cautiously when comparing patient groups. As noted, confirmation of a lack of bias has important implications for both clinicians and researchers.

The findings of this study suggest that a thorough development process can limit the risk of inherent biases in a measurement tool. We attribute the remarkably low level of DIF and DTF detected in PRISM-CC to rigorous application of a number of practices, many of which are recommended in guidelines for developing patient-reported measures [28, 29]. We propose that avoiding DIF and DTF in measures is best addressed at a measure’s conceptual and development phase. We further suggest that collaborations across countries and disciplines can lead to the development of stronger instruments. The PRISM-CC is unusual in that it was simultaneously developed in two languages by a multidisciplinary (nursing, occupational therapy, physiotherapy, and health services researchers) and multilingual (English, Swedish, Portuguese, and Mandarin first language speakers) team [23, 31]. Translatability was extensively used to inform item wording and selection. Further, patients were engaged in the item development and selection process. Cognitive interviewing was used to identify differences in patient interpretation, which contributed to item revision and selection.

In this study, DIF/DTF was tested for four demographic variables (age, education, gender and number of conditions) and two languages. However, other variables have also been found to associate with self-management behaviours. For example, self-efficacy underpins many self-management interventions and has been shown to be strongly associated with self-management [34, 37]. Likewise, impaired functional ability such as decreased strength or sensory impairments [71], disease progression, years living with a condition [33], cognitive impairment [35], family and social support [33, 72, 73], health literacy [34, 74, 75], or mental health [76–78] have all been related to self-management. Future research to better understand if these variables are associated with bias in PRISM-CC scores would further strengthen confidence in its use.

Limitations in the available data may have impacted this study’s ability to detect meaningful DIF and DTF. For example, the assessment of DIF and/or DTF by disease duration and type of condition was not possible. This, along with different categorizations of education and number of conditions should be a priority for future research since it may be important for clinical practice and research. As previously noted, the Swedish sample did not allow assessment of DIF and DTF by age. Using a close-to representative sample of older adults age 72 − 23, meant the number participants with severe self-management challenges may have been underpowered to detect DIF in this subgroup—a limitation that future clinical studies should also address. Despite this analytical limitation, the findings are generalizable to a similar cohort of older adults in Sweden. Findings from the large English dataset, a convenience sample that specifically recruited participants living with a chronic condition, may be more similar and generalizable to clinicial populations.

The Swedish and English samples differed considerably in their demographic and chronic disease attributes, which is both a limitation and a strength. The key limitation is that isolation of DIF/DTF by language is challenging. We addressed this by first ensuring there was no meaningful DIF within either sample by age, education and number of conditions before pooling to test for language differences. An alternative approach woud be to select matched samples from the two data sources before testing for DIF/DTF, but there was not sufficient overlap in subject characteristics to permit this. As a crude alternative, a sensitivity analyses where only participants > 40 years of age in the English data were pooled with the Swedish data yielded nearly identical results. The assessment of DIF and DTF between the English and Swedish samples is a key strength, however, if framed as global assessment covering differences by language, culture, sciodemographic and health attributes. The fact that no meaningful DIF or DTF was found in two considerably different samples adds strength to the findings that there is little DIF or DTF in the PRISM-CC items or domain scores.

## Conclusion

The absence of meaningful DIF and DTF in the PRISM-CC underscores its significance as a valuable tool for assessing self-management difficulty. It demonstrates its utility for application across diverse demographic groups and languages. Combined with previous evidence of its strong psychometric properties in Swedish and English, the PRISM-CC is a promising instrument for research and clinical practice.

## Data Availability

The dataset generated and analysed during the current study is not publicly available because participant consent included restrictions on the use of the data due to patients’ privacy concerns. Limited availability is possible. Researchers wishing for information may contact the corresponding author Åsa Audulv.

## Abbreviations

DIF: Differential item functioning
DTF: Differential test functioning
HAI: Healthy aging initiative
IRT: Item response theory
NA: Not applicable
PRISM-CC: the Patient Reported Iinventory of Self-Management of Chronic Conditions
TEDSS: Taxonomy of Everyday Self-management Strategies Framework
TCC: Test characteristic curves
